# The Connection-set Algebra: a formalism for the representation of connectivity structure in neuronal network models, implementations in Python and C++, and their use in simulators

**DOI:** 10.1186/1471-2202-12-S1-P80

**Published:** 2011-07-18

**Authors:** Mikael Djurfeldt

**Affiliations:** 1 PDC, KTH, S-100 44 Stockholm, Sweden; 2 INCF, KI, S-171 77 Stockholm, Sweden

## 

The connection-set algebra (CSA) [1,2] is a novel and general formalism for the description of connectivity in neuronal network models, from its small-scale to its large-scale structure. It provides operators to form more complex sets of connections from simpler ones and also provides parameterization of such sets.

The CSA is expressive enough to describe a wide range of connectivities and can serve as a concise notation for network structure in scientific writing. CSA implementations allow for scalable and efficient representation of connectivity in parallel neuronal network simulators and could even allow for avoiding explicit representation of connections in computer memory. The expressiveness of CSA makes prototyping of network structure easy.

Here, a Python implementation [4] of the connection-set algebra is presented together with its application to describing various network connectivity patterns. In addition, it is shown how CSA can be used to describe network models in the PyNN [5] and NineML [6] network model description languages.
